# A novel complement C3 inhibitor CP40-KK protects against experimental pulmonary arterial hypertension via an inflammasome NLRP3 associated pathway

**DOI:** 10.1186/s12967-023-04741-z

**Published:** 2024-02-16

**Authors:** Lei Dai, Yu Chen, Jinhua Wu, Zhen He, Yueqi Zhang, Wenjun Zhang, Yang Xie, Hesong Zeng, Xiaodan Zhong

**Affiliations:** 1 Division of Cardiology, Department of Internal Medicine, Tongji Hospital, Tongji Medical College, Huazhong University of Science and Technology, Wuhan, 430030 China; 2 Hubei Provincial Engineering Research Center of Vascular Interventional Therapy, Wuhan, 430030 Hubei China; 3 Department of Gastroenterology, The People’s Hospital of Guangxi Zhuang Autonomous Region, Nanning, 530000 Guangxi China

**Keywords:** Pulmonary arterial hypertension, Complement C3, CP40-KK, NLRP3, Proinflammatory cytokine

## Abstract

**Background:**

Pulmonary arterial hypertension (PAH) is a severe cardiopulmonary disease characterized by complement dependent and proinflammatory activation of macrophages. However, effective treatment for complement activation in PAH is lacking. We aimed to explore the effect and mechanism of CP40-KK (a newly identified analog of selective complement C3 inhibitor CP40) in the PAH model.

**Methods:**

We used western blotting, immunohistochemistry, and immunofluorescence staining of lung tissues from the monocrotaline (MCT)-induced rat PAH model to study macrophage infiltration, NLPR3 inflammasome activation, and proinflammatory cytokines (IL-1β and IL-18) release. Surface plasmon resonance (SPR), ELISA, and CH50 assays were used to test the affinity between CP40-KK and rat/human complement C3. CP40-KK group rats only received CP40-KK (2 mg/kg) by subcutaneous injection at day 15 to day 28 continuously.

**Results:**

C3a was significantly upregulated in the plasma of MCT-treated rats. SPR, ELISA, and CH50 assays revealed that CP40-KK displayed similar affinity binding to human and rat complement C3. Pharmacological inhibition of complement C3 cleavage (CP40-KK) could ameliorate MCT-induced NLRP3 inflammasome activity, pulmonary vascular remodeling, and right ventricular hypertrophy. Mechanistically, increased proliferation of pulmonary arterial smooth muscle cells is closely associated with macrophage infiltration, NLPR3 inflammasome activation, and proinflammatory cytokines (IL-1β and IL-18) release. Besides, C3a enhanced IL-1β activity in macrophages and promoted pulmonary arterial smooth muscle cell proliferation in vitro.

**Conclusion:**

Our findings suggest that CP40-KK treatment was protective in the MCT-induced rat PAH model, which might serve as a therapeutic option for PAH.

**Supplementary Information:**

The online version contains supplementary material available at 10.1186/s12967-023-04741-z.

## Introduction

Pulmonary arterial hypertension (PAH) is a progressive disorder characterized by vasoconstriction and remodeling of the small pulmonary arteries, leading to elevated pulmonary artery pressure, right heart failure, and eventually death [1–3]. Disease pathogenesis is driven by pathologic remodeling and vasoconstriction of the pulmonary arteries, caused by the excessive proliferation and migration of vascular wall cells, and perivascular inflammation [4]. Significantly increased macrophages were found in pulmonary vascular lesions, which secrete vascular endothelial growth factor (VEGF) and platelet derived growth factor (PDGF), contributing to pulmonary vascular endothelial cell damage and apoptosis, and promoting smooth muscle cell migration and proliferation [5].

Present estimates suggest that 1% of the world population and up to 10% of persons aged > 65 years have pulmonary hypertension [6]. The current treatment of PAH mainly consists of the single or combined administration of phosphodiesterase-5 inhibitors, endothelin-receptor antagonists, soluble guanylate cyclase stimulators, and compounds targeting the prostacyclin pathway [2]. Recently, sotatercept, a novel first-in-class activin signaling inhibitor, has been shown to improve clinical outcomes in a randomized controlled trial  [7]. Despite advances in understanding of the pathophysiology and the management of PAH, effective treatment for this life-threatening disease is still lacking, therefore, new strategies are urgently required [8, 9].

The complement system acts as a first line of defense against injurious stimuli and invading pathogens. It has three differential activating cascades, containing the classical, alternative, and lectin pathways, which converge at the level of C3 cleavage. The activation leads to the production of opsonin (C3b), the membrane attack complex (C5b-9), and anaphylatoxins (C3a and C5a) [10, 11]. Bauer EM et al. have shown that significant C3 fragment deposition (C3d) was found in lung sections from idiopathic PAH patients and chronic hypoxia-induced PAH mice, furthermore, the indices of PAH got significantly ameliorated in C3 knockout mice [12]. Complement activation, specifically that of the alternative pathway, plays a critical role in the early inflammatory and proliferative responses observed in pulmonary hypertension (PH) [13]. Complement activation was also noted to be important in the recruited macrophages that contributed to the PH process [14]. Fibroblast-released small extracellular vesicles served as critical mediators of complement-induced perivascular/microenvironmental inflammation in PH [15]. However, a question regarding the clinical translational aspect, such as whether in vivo intervention by specific complement C3 inhibitor could be amendable for PAH, remains open.

The compstatin family, a group of short cyclic peptides, can selectively and strongly bind to C3 and its fragments, preventing complement activation [16]. The most potent derivative, CP40, has been proved beneficial in various diseases, such as unilateral ureteral obstruction [17], kidney allograft survival [18], COVID-19 immunothrombosis [19], paroxysmal nocturnal hemoglobinuria [20], chronic inflammatory conditions in dialysis [21], intra- and extravascular hemolysis of red blood cells [22], malarial anemia [23], periodontitis [24], xenoreaction [25], hemorrhagic shock [26], C3 glomerulopathy [27], etc. Moreover, the immune system is not weakened in healthy adult non-human primates, and susceptibility to infections is not affected after systemic C3 inhibition [28]. These splendid characteristics potentiate CP40 as a good candidate for clinical application. Surprisingly, Berger et al. recently demonstrated that the addition of two residues of lysine to CP40 (CP40-KK), could not only improve the peptide’s solubility but also increase the binding affinity for C3b while retaining its inhibitory potency. These pharmacokinetic profiles have been tested in vitro and in vivo in non-human primates as well [29].

Interestingly, accumulating data demonstrate that the inflammasome is highly regulated by complement and is necessary during the resolution of tissue injury or disease [30]. Recent studies have shown that the NLRP3 inflammasome is activated in the lung macrophages in animal models of PH and that inhibiting the inflammasome ameliorates monocrotaline (MCT) or hypoxia-induced PH [31–34]. However, the detailed molecular mechanism (s) by which complement activation causes PAH remains elusive. To this end we hypothesize that complement activation could provoke NLRP3 inflammasome cascade, subsequently induces proinflammatory cytokines (IL-1β and IL-18), and could further promote endothelial dysfunction and vascular remodeling in pulmonary arterioles, finally results in PAH.

Thus, in the current study, we aim to confirm the role of complement provoked NLRP3 activation in the occurrence and progression of PAH and evaluate the curative efficacy of the newly identified selective C3 inhibitor CP40-KK in the MCT-induced rat PAH model.

## Materials and methods

### Reagents

Monocrotaline (Sigma-Aldrich, St. Louis, MO) was dissolved in 1 mol/L HCl and then titrated to pH 7.4 with 1 mol/L NaOH with the final concentration of 20 mg/mL. Complement inhibitor compstatin analog CP40-KK (dTyr-Ile-[Cys-Val-Trp(Me)-Gln-Asp-Trp-Sar-His-Arg-Cys]-mIle-Lys-Lys-NH2; 2.0 kDa) was produced by solid-phase peptide synthesis (GL Biochem Shanghai, China) and was dissolved in sterilized 0.9% NaCl solution with a concentration of 2 mg/ml. Complement C3 was purchased from ProSpec (Human C3:PRO-2684; Rat C3:PRO-2706). Other reagents used in the current study were shown in the supplement.

### Animal models and experimental protocols

This study was performed in compliance with the Guide for the Care and Use of Laboratory Animals (NIH Publication No. 85–23, revised 1996. All animal procedures and protocols were approved by the Ethics Committee of Tongji Medical College, Huazhong University of Science and Technology (Wuhan, China). Adult male Sprague–Dawley rats (180–250 g) were obtained from the Laboratory Animal Services Centre, Tongji Medical College. All rats were provided food and water ad libitum and housed under a 12:12 h light–dark cycle at 18–22 °C and 50–70% humidity.

In the current study, two animal experiments were conducted successively. 30 rats were randomly divided into two groups (control group and MCT group, n = 15 per group) in the first experiment. MCT group rats received a single intraperitoneally injection of MCT (40 mg/kg) on day 1 to induce PAH while control group rats received an equivoluminal saline injection intraperitoneally. For the second animal experiment, the rats were assigned into four groups (control group, MCT group, CP40-KK group, and MCT + CP40-KK group, n = 15 per group). The control group was given the same volume of saline subcutaneously while the CP40-KK group rats only received CP40-KK (2 mg/kg) by subcutaneous injection from day 15 to day 28 continuously. The rats in the MCT group and MCT + CP40-KK group were received a single intraperitoneally injection of MCT (40 mg/kg) on day 1 and subcutaneously injected with either an equal volume of saline or CP40-KK (2 mg/kg) at day 15 to day 28 continuously. The dose selection of MCT and CP40-KK in our study was established based on previous studies [17, 35, 36].

### Hemodynamic measurement and right ventricular hypertrophy index analysis

Four weeks after the MCT injection, the experimental rats were weighed and anesthetized by intraperitoneal injecting pentobarbital sodium (40 mg/kg). After being anesthetized, rats were fixed in the supine position. A 3F polyethylene catheter filling with heparin was introduced into the right ventricle (RV) through the right jugular vein and the right atrium. Right ventricle systolic pressure (RVSP) was assessed by a pressure transducer and PowerLab system (AD Instruments, Australia). Pressure recordings were measured over a 2 min period and analyzed with Powerlab Pro Software (AD Instruments, Australia). After the hemodynamic study, the rats were euthanized by an overdose of anesthesia, and their lungs and hearts were dissected for further evaluation. The dry weights of the RV and left ventricle (LV) + septum were measured to calculate the RV/(LV + S) ratio and the RV / body weight (RV/BW). The left lung was fixed in 4% paraformaldehyde, and the right lung was snap-frozen in liquid nitrogen and stored at − 80 °C for further analysis [37, 38].

### Morphological investigation

Fixed lung samples were further fixed in 4% buffered paraformaldehyde for 24 h, embedded in paraffin, and processed for sectioning at 5 μm thick. Hematoxylin and eosin (H&E) staining were performed on paraffin-fixed lung tissue slides. The percent medial wall thickness (WT%) and percent medial wall area (WA%) were used to assess the extent of pulmonary vascular remodeling. WT% = (external diameter − internal diameter)/(external diameter) × 100%; WA% = (external area − internal area)/external area × 100%. All data were obtained and analyzed in a blinded manner [39].

### Isolation and culture of primary rat pulmonary arterial smooth muscle cells (PASMCs)

PASMCs were isolated from rat distal pulmonary arteries and then cultured in Dulbecco’s modified Eagle’s medium (DMEM) containing 20% fetal bovine serum as previously described [40].

### EdU assay

Cells seeded in the 24-well plate were labeled with 10 μmol/L of EdU (Meilun, Dalian, China) for 2 h at 37 ℃. Subsequently, cells were fixed in 4% of paraformaldehyde for 20 min and incubated in phosphate-buffered saline (PBS) containing 0.5% of Triton- X100 for 15 min. After being washed three times with PBS, 150 μL of dying solution was applied per well for 30 min and nuclei staining with Hoechst 33,342 for 10 min away from light. The proliferating cells were labeled with green fluorescence, with a maximum excitation wavelength of 491 nm and a maximum emission wavelength of 518 nm for 488-Azide. The proliferative rate = EdU-positive cells/Hoechst-positive cells.

### Cell culture and treatment

RAW264.7 cells were provided by the Hubei Key Laboratory of Genetics and Molecular Mechanism of Cardiologic Disorders, Huazhong University of Science and Technology (China), and cultured in DMEM containing 10% fetal bovine serum at 37 ℃ and 5% CO_2_. RAW264.7 cells were incubated with 50 nM C3a for 12 h and then were washed twice with PBS. The cells were incubated with serum-free DMEM for 12 h and the supernatants were collected. After pretreatment with 100 ng/ml of IL-1RA for 1 h, we added the supernatants from the control or C3a group to PASMCs respectively for 24 h.

### Immunohistochemistry and immunofluorescence

After hemodynamic measurements, the lungs were perfused with cold phosphate-buffered saline (PBS) and fixed in 4% formaldehyde for 24 h. The whole lungs were embedded in paraffin, and cross-sections (5 μm) were prepared. Paraffin sections were used for immunostaining. For immunohistochemistry, lung sections were initially de-paraffinized, rehydrated, and boiled in a pressure cooker containing a citric acid buffer (pH 6.0) for 20 min to retrieve antigens. Then the slides were blocked with 5% BSA for 1 h and incubated with primary antibodies overnight at 4 °C and with corresponding secondary antibodies for 30 min at room temperature. The primary antibody to αSMA (1:100, Abclonal) and Galectin 3 (1:100, Servicebio) were used. Immunolabels were detected with 3, 3-Diaminobenzidine (DAB), after which the nuclei were counterstained with hematoxylin. Slides were inspected under an optical microscope (Olympus BX61, Tokyo, Japan) at 200 × and 400 × magnification. For quantification analysis, the mean integrated optical density (IOD) for immunohistochemistry was determined using Image J software (National Institutes of Health, Bethesda, MD) [41].

For immunofluorescence, the lung slides were prepared as described in histological analysis till the incubation of secondary antibodies. Corresponding fluorescently labeled secondary antibodies (anti-mouse IgG FITC: 1:200, anti-rabbit IgG-CY3 red: 1:200) were added for 60 min and sections were rinsed twice in PBS. Slides were covered with vecta shield mounting medium containing nuclear stain DAPI (Vector Laboratories, CA, USA). Specimens were analyzed on a Leica SP5 confocal microscope [42].

### Western blotting

Proteins were collected from the fresh frozen-rat lung tissues using RIPA lysis buffer. Protein concentration was quantified using a BCA reagent. Equal amounts of protein were electrophoretically separated on 10% SDS polyacrylamide gel, transferred to PVDF membranes, and probed with desired primary antibodies overnight at 4 °C. Membranes were then washed with TBST and incubated with anti-mouse IgG (1:5000) or anti-rabbit IgG (1:5000) horseradish peroxidase conjugated antibodies as indicated. Blots were developed with the enhanced chemiluminescence system. To compare and quantify levels of proteins, the density of each band was measured using Image J software. Equal loading for total cell or tissue lysates was determined by β-Actin western blot [43, 44].

### ELISA

CP40-KK and its control peptide were dissolved in coating buffer at different concentration and then added to wells of ELISA plate as designed. The coated plate was incubated at 4 °C overnight. Then the plate was incubated with plasma with the indicated dilution at 37 °C for 2 h. Next, the plate was incubated with the C3 antibody (1:2000, MP biomedicals, Irvine, CA, USA) at 37 °C for 2 h. Then the plate was washed for 3 times and incubated with corresponding HRP conjugated secondary antibodies (1:2000, Abcam, Cambridge, UK) at 37° for 1 h. Finally, TMB substrate was added to each well for indicated time at 37 °C and then the stop solution was added to stop the TMB reaction. OD values were measured at 450 nm using a microplate reader (Bio-tek, Vermont, USA). The coating buffer, wash buffer, TMB substrate, and stop solution were all bought from Boster Biological Technology (Wuhan, China).

### CH50 assay

The total functional complement was determined with a CH50 assay based on the modified method of Mayer. The 50% haemolytic standard tube was made by adding 0.3 ml 2% sheep erythrocytes (Chundubio, Wuhan, China) to 1.2 ml distilled water and then diluting with equivoluminal veronal buffer saline (Leagene, Beijing, China). The sheep erythrocytes were sensitized by adding a designated volume of a predetermined concentration of rabbit haemolytic serum (Solarbio, Beijing, China) in veronal buffer saline. The plasma of rats was first prediluted in veronal buffer saline at 1/5, 1/10, and 1/20. At every dilution, ten different volumes (20, 30, 40, 50, 60, 70, 80, 90, 100, and 0 µl) of prediluted plasma of rats were firstly mixed with corresponding volumes of veronal buffer saline in ten tubes and then all ten tubes were added 200 µl sensitized sheep erythrocytes respectively. The OD values of supernatant liquid in the ten tubes were measured at 541 nm and compared with that of the 50% haemolytic standard tube to get the most similar value. The total haemolytic complement (CH50) = dilution ratio / the quantity of plasma needed to induce 50% lysis of sheep erythrocytes [45–47].

### Surface plasmon resonance (SPR)

Before the experiment, Sensor Chip COOH and 200μL EDC /NHS solution should be prepared. Prepare all buffers containing Running Buffer HEPES, Immobilization Buffer, and Regeneration Buffer. Then, dilute the human complement C3 (rat complement C3、mouse complement C3) to 62.5 μg/mL in an immobilization buffer. The activator is prepared by mixing 400 mM EDC and 100 mM NHS immediately prior to injection. Then inject to sample to reach a capture level of about 4800 RU. The chip is deactivated by 1 M Ethanolamine hydrochloride for 240 s. Dilute compstatin (CP40-KK) with the same running buffer to 5 concentrations. Compstatin (CP40-KK) is injected into the flow cell of the channel for an association of 240 s, followed by 480 s dissociation. The association and dissociation processes are all handled in the running buffer. Repeat 5 cycles of analyte according to analyte concentrations in ascending order. The analysis software used in this experiment is: TraceDrawer (Ridgeview Instruments ab, Sweden), and was analyzed by the One To One analysis model.

### Statistical analysis

One-way ANOVA and the Student’s *t*-test were used to evaluate any variations in outcomes between different groups as appropriate (GraphPad Prism 9.0, GraphPad Software, Inc.; and Excel software, Microsoft). The data was summarized as the mean ± SEM (Standard Error of Mean). A *P*-value < 0.05 was considered as statistically significant.

## Results

### C3a was significantly upregulated in the plasma of MCT-induced PAH rats

**Fig. 1:**
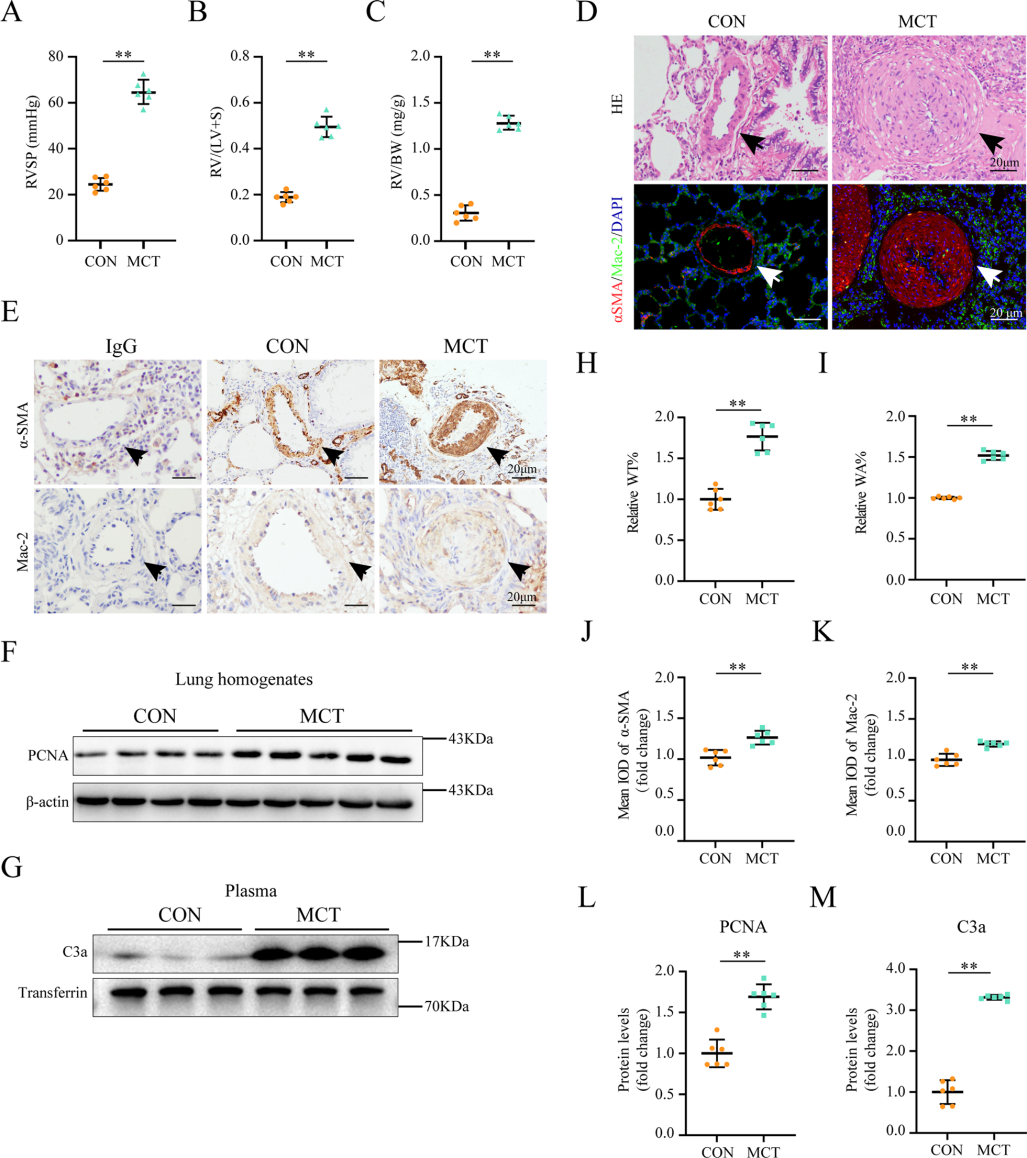
C3a was significantly upregulated in the plasma of MCT-induced PAH rats **A**–**C** Right ventricular systolic pressure (RVSP), right ventricular hypertrophy indexes (RV/LV + S), and RV/body weight (BW) increased significantly after MCT treatment (n = 6 per group). **D**,**H**–**I** Representative HE staining images of rat lungs from the two groups (**D**, upper) and graphs reflecting the percentage of medial wall thickness WT% and vessel wall area WA% (**H**, **I**; n = 6 per group). **E**,**F**,**J**,**L** Pulmonary vascular remodeling was aggravated by MCT. Representative immunohistochemical staining images of α-SMA (**E**, upper) and graphs summarizing results (**J**; n = 6 per group). Representative western blots and quantification of PCNA protein levels in lung homogenates (**F**,**L**; n = 6 per group). (**D**, lower and **E**, lower**)** representative immunofluorescence and immunohistochemistry staining for Mac-2 of the distal pulmonary arteries exposed to MCT (n = 6 per group). **G**,**M** Representative Western blots and quantification of C3a protein levels in the rat plasma (n = 6 per group). Scale bar: 20 μm (**D**,**E**); MCT: monocrotaline; RV: right ventricle; LV: left ventricle; S: septum; mean ± SEM; comparisons between the control and MCT groups made by student *t* test; *P < 0.05, **P < 0.01

We conducted the hemodynamic and histological examinations at 4 weeks post MCT injection. As shown in Fig. 1A–C, RVSP after the 4 weeks of MCT treatment went up (63.03 ± 4.19 *vs.* control group 28.00 ± 3.55 mmHg, *P* < 0.01). We performed H&E and immunohistochemistry staining to evaluate the pathological changes in the small pulmonary arteries (50–200 mm). H&E staining showed that pulmonary small arteries from control group rats had thinner vascular walls and larger vessel lumen than those from MCT groups (Fig. 1D). Furthermore, pulmonary arterial wall thickness index (WT% and WA%) remarkably increased in MCT groups compared with those in control group (*P* < 0.01 and *P* < 0.01, respectively, Fig. 1H, I). The expression of α-SMA and Mac2 were elevated significantly in the walls of small pulmonary arteries of the MCT groups compared with those in the control group (*P* < 0.01 and *P* < 0.01, respectively, Fig. 1D, E, J, and K). PCNA expression was moderately increased in the lung homogenates of the MCT group (*P* < 0.01, Fig. 1F, and L). All these results indicated that PAH is well established in rats after MCT injection.

Interestingly, C3a was significantly upregulated in the plasma of MCT-induced PAH rats (*P* < 0.01, Fig. 1G, M). In the lung homogenates, MCT induced an elevated expression level of p-P65/p65, and the NF-κB nuclear translocation resulted in the increased expression of key proinflammatory component NLRP3 and caspase-1. We hypothesized that C3a induced a pro-inflammatory state via NLRP3 inflammasome in macrophages of PAH rats.

### New analogs of the C3 inhibitor compstatin (CP40-KK) could bind not only human C3 but also rat C3

**Fig. 2:**
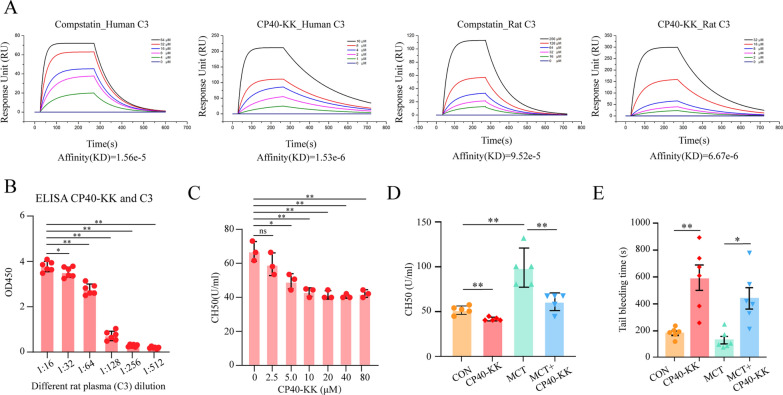
New analogs of the C3 inhibitor compstatin (CP40-KK) could bind not only human C3 but also rat C3 **A** Representative sensorgram showing the binding of CP40-KK to immobilized human C3 and rat C3. **B** The ELISA based approach showed the direct binding of rat C3 and CP40-KK. **C**, **D** The CH50 test (Mayer method) was applied to measure rat complement C3 function. **E** The tail bleeding time was measured in four groups. Multiple comparisons made by one-way ANOVA; *P < 0.05,  **P < 0.01

Compstatin inhibits human complement by interacting with human C3 and inhibiting its cleavage by C3 convertase in a species-specific manner. However, new analogs of the C3 inhibitor compstatin (CP40-KK) with increased solubility and improved pharmacokinetic profile [29]. Human complement C3 captured on the COOH chip could bind compstatin and CP40-KK with an affinity constant of 15.6 μM and 1.53 μM as determined in the LSPR assay. Rat complement C3 captured on the COOH chip could bind compstatin and CP40-KK with an affinity constant of 95.2 μM and 6.67 μM as determined in the LSPR assay **(**Fig. 2A**)**. CP40-KK displayed stronger binding affinity to complement C3 than compstatin. CP40-KK could bind rat complement C3, but the binding affinity is slightly lower than that for CP40-KK to human complement C3.

The ELSA based approach was also used to show the direct binding of rat C3 and CP40-KK. As shown in Fig. 2B, the OD450 values peaked at 1:16 of plasma dilution and declined further as the dilution went up. It indicated a specific binding of the CP40-KK and rat C3. In addition, we have done the complement function assay, the CH50 test (Mayer method) in vitro and ex vivo. We applied different concentrations of CP40-KK (0, 2.5, 5.0, 10, 20, 40 µM) to the mixed wild-type SD rat plasma to block the C3 activation. As we expected the concentration of CP40-KK correlates negatively with the CH50 value, which conveyed the message that CP40-KK blocked rat C3 function in a dosage-dependent manner **(**Fig. 2C**)**. Moreover, the CH50 results from rat plasma samples of the PAH experiment groups stand in line with our hypothesis, MCT group has the highest average CH50 value, indicating obvious C3 activation, while CP40-KK treatment could reverse it, which means CP40-KK worked in vivo in rats as well **(**Fig. 2D**).** Tail bleeding time after 2 weeks of CP40KK treatment in different groups were also analyzed, CP40KK could significantly prolong the tail bleeding time in the rats **(**Fig. 2E**).**

### Selective complement C3 inhibitor CP40-KK ameliorates indices of PAH in an established rat PAH model

**Fig. 3:**
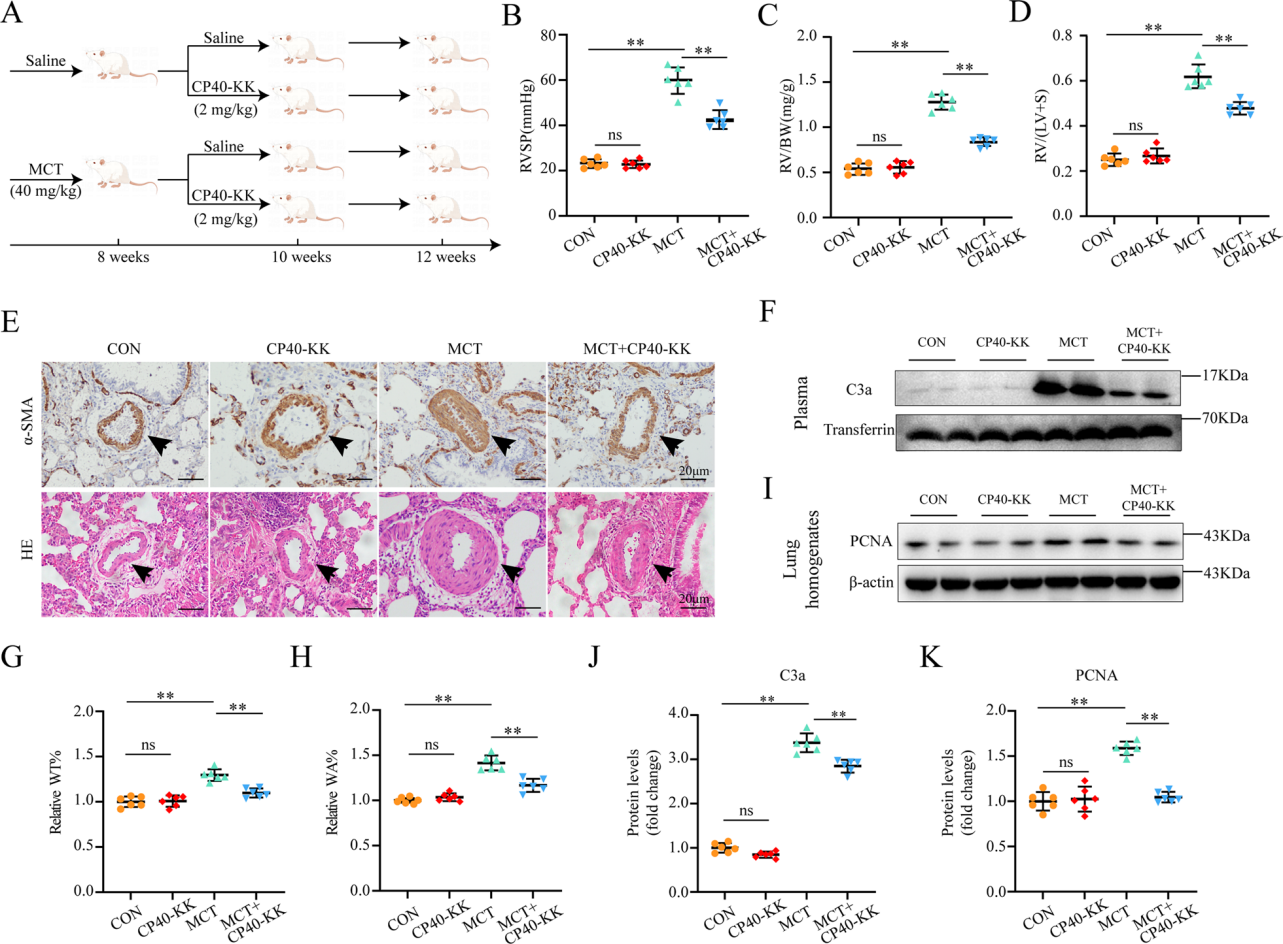
Selective complement C3 inhibitor CP40-KK ameliorates indices of PAH in an established rat PAH model **A** Schematic diagram of animal experiment procedure. PAH was induced by MCT at the dose of 40 mg/kg at day 1. Treatment with CP40-KK 2 mg/kg/day by subcutaneous injection at day 15 to day 28 continuously. **B**–**D** Right ventricular systolic pressure (RVSP), right ventricular hypertrophy indexes (RV/LV + S) and RV/body weight (BW) were decreased by CP40-KK in MCT-induced PAH rats (n = 6 per group). **E**,**G**–**H** Representative HE staining images of rat lungs from all four groups **E** and graphs reflecting the percentage of medial wall thickness WT% and vessel wall area WA% (**G**,**H**; n = 6 per group). **E**,**I**,**K** Pulmonary vascular remodeling was ameliorated by CP40-KK in MCT-induced PAH rats. **E** Representative immunohistochemical staining images of α-SMA from all four groups.** I**,**K **Representative western blots and quantification of PCNA protein levels in lung homogenates (n = 6 per group). **F**,**J** Representative Western blots and quantification of C3a protein levels in the rat plasma (n = 6 per group). Scale bar: 20 μm (**E**,**F**); MCT: monocrotaline, RV: right ventricle, LV: left ventricle, S: septum; mean ± SEM; Multiple comparisons made by one-way ANOVA; *P < 0.05, **P < 0.01

**Fig. 4:**
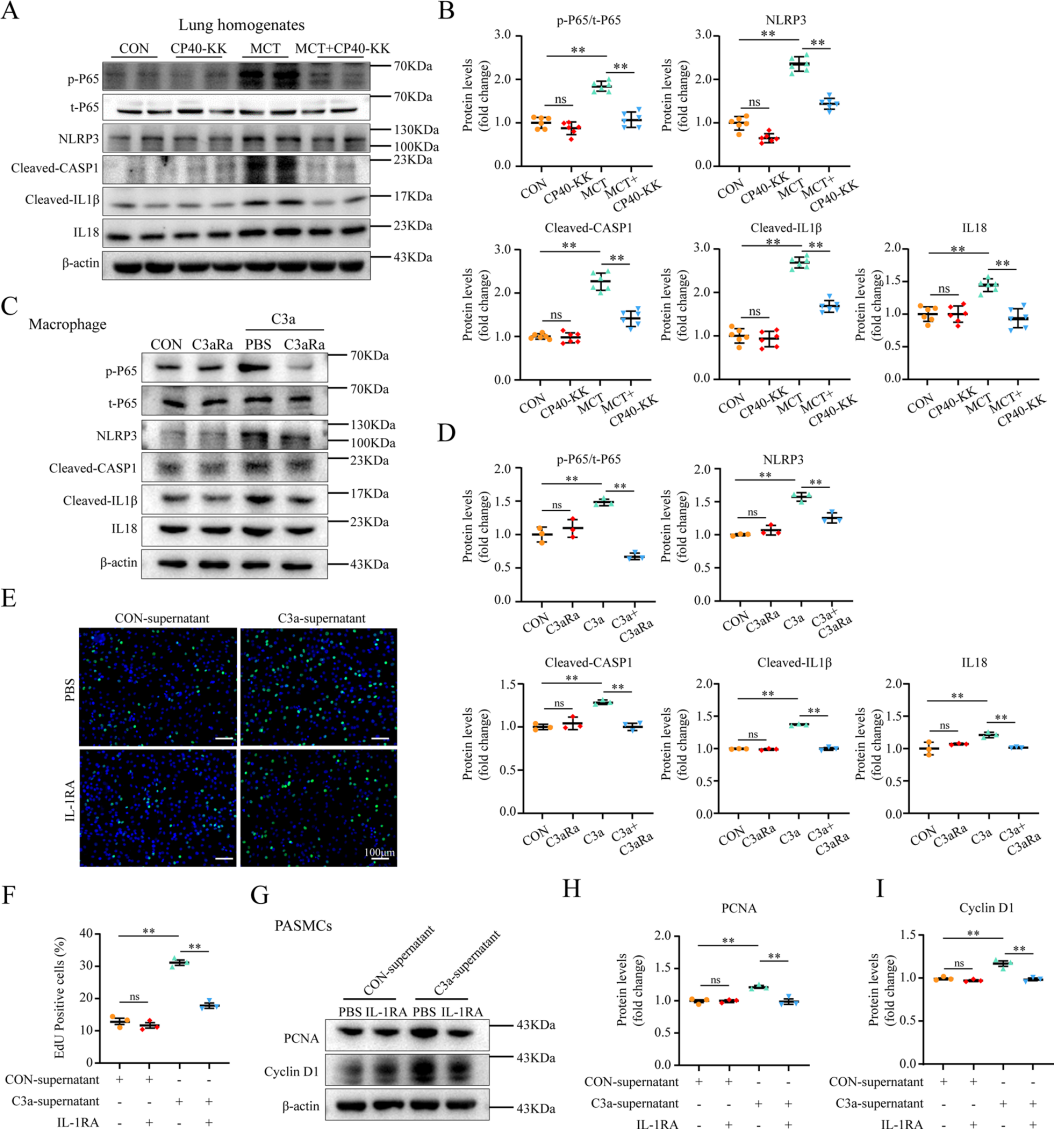
C3a induces a pro-inflammatory state via NLRP3 inflammasome in macrophage of PAH rats **A**–**B** Increased levels of the p-P65/P65, NLRP3, Cleaved-CASP1, Cleaved-IL1β, and IL18 in lung homogenates of the MCT group as compared to the MCT + CP40-KK group. Representative immunoblot **A** and graphs summarizing results (B; n = 6 per group). Representative Western blots **C** and quantification of p-P65/P65, NLRP3, Cleaved-CASP1, Cleaved-IL1β, IL18 in RAW264.7 after treatment with C3a (50 nM) for 24 h (**D**; n = 3 per group). **E**, **F** Representative EdU assay determined the proliferative rate in PASMCs under the CON-supernatant or C3a-supernatant condition plus treatment with PBS or IL-1RA for 24 h (n = 3 per group). Representative Western blots **G** and quantification of PCNA and Cyclin D1 in PASMCs under the CON-supernatant or C3a-supernatant condition plus treatment with PBS or IL-1RA for 24 h (H,I; n = 3 per group). Scale bar: 100 μm **E**; mean ± SEM; Multiple comparisons made by one-way ANOVA; *P < 0.05, **P < 0.01

Two weeks of CP40-KK treatment was executed, and it could significantly ameliorate the above-mentioned PAH-related indices, such as RVSP, RV / (LV + S) and RV / BW ratios, pulmonary parietal wall thickness indices (WT% and WA%), whereas CP40-KK only group has no significant effect in comparison with saline only group (Fig. 3B–D, G, and H). Immunohistochemically, the expression of α-SMA was reduced significantly in the walls of small pulmonary arteries of the MCT + CP40-KK group compared with those in the MCT group (Fig. 3E). The plasma C3a western blot results showed that CP40-KK could significantly inhibit the C3 activation ex vivo in the rats (Fig. 3F, J). PCNA expression was decreased in the MCT + CP40-KK group compared with those in the MCT group (Fig. 3I, K). With the help of western blot, we found CP40-KK could significantly block NF-κB subunit p65 phosphorylation and NLRP3 inflammasome activation, which in turn reduced the production of proinflammatory cytokines, such as IL-1β and IL-18 finally resulting in decreased vascular smooth muscle cell proliferation and improved pulmonary arterial remodeling and survival (Fig. 4A–B). Surprisingly, CP40-KK has no effect on lipid metabolism or oxidative stress (Additional file 1: Figures S1, S2 in the Additional file materials). All these results indicated the therapeutic effect of CP40-KK in an established rat PAH model.

### C3a induces a pro-inflammatory state via NLRP3 inflammasome in macrophage of PAH rats

We found C3a stimulated macrophages to produce cleaved-IL1β and evaluated the expression levels of NF-κB subunit p65 phosphorylation, cleaved-caspase-1, and NLRP3. Addition of 1 mM C3aRa reduced the cellular levels of NLRP3, cleaved-caspase-1, cleaved-IL1β, and IL18 **(**Fig. 4C–D**)**.

### Macrophage-derived IL1β promotes PASMC proliferation

**Fig. 5:**
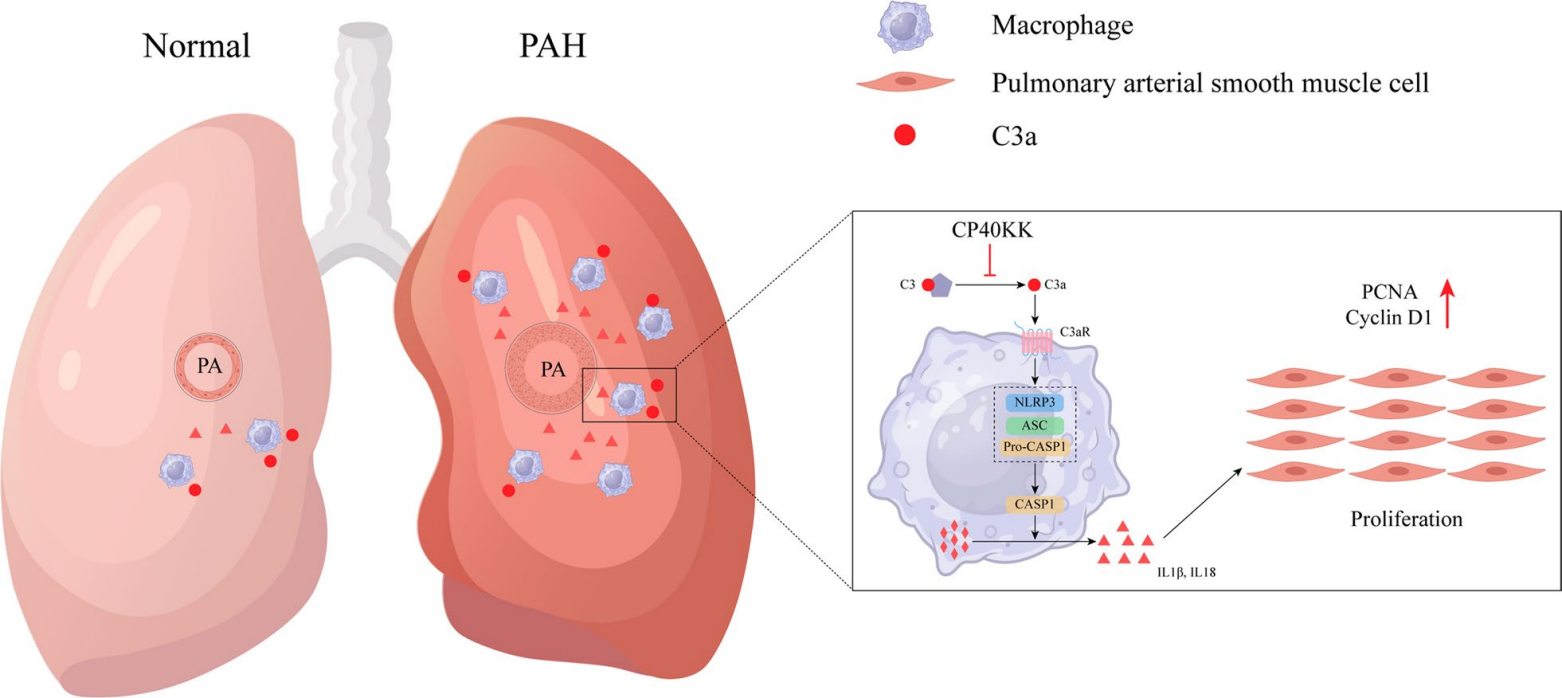
Schematic Representation of the mechanism of CP40-KK in the therapy of PAH Pulmonary arterial hypertension (PAH) is triggered by macrophage–NLRP3 activation and can be blocked by CP40-KK. Proposed mechanism of NLRP3 activation in PAH: the activation of the NLRP3 inflammasome pathway in rat lung is triggered by complement C3a in plasma. NLRP3 inflammasome assembles with apoptosis-associated speck-like protein (ASC), leading to release of cleaved-IL1β and IL18. Cleaved-IL1β and IL18 could promote PASMCs proliferation, contributing to pulmonary vascular remodeling. This study proposed one therapeutic approach to cease the inflammatory process: CP40-KK, a selective complement C3 inhibitor, preventing complement C3 activation. ASC: apoptosis-associated speck-like protein

C3a stimulated macrophages to produce and secret cleaved-IL1β in the supernatant. Additionally, we used the supernatant to culture PASMCs and evaluated the effects on proliferation using WB and an EdU assay. EdU assay revealed that the number of EdU-positive PASMCs was significantly increased upon the addition of macrophage-conditioned media and this effect was reversed by IL-1RA. PCNA and Cyclin D1 expression were decreased in the C3a-supernatant + IL-1RA group compared with those in the C3a-supernatant + PBS group (Fig. 4E–I). Surprisingly, CP40-KK, a selective complement C3 inhibitor could protect against the established rat PAH model via inflammasome NLRP3 suppression **(**Fig. 5**)**.

## Discussion

The pathobiology of PAH is complex and incompletely understood, despite the recently achieved advances, effective treatments are still lacking. In the current study, we confirmed the vital role of the complement system, especially, its key component C3a, in PAH. In detail, in the smooth muscle cell layer of the MCT-induced PAH rat pulmonary small artery, C3a could promote subsequent inflammasome NLRP3 activation and release proinflammatory cytokines (IL-1β and IL-18) resulting in vascular remodeling and finally pulmonary arteriole obstruction. Furthermore, we were the first to show that the CP40-KK, a newly identified analog of the selective C3 inhibitor CP40, could ameliorate the established PAH in the MCT-induced rat model, which therefore, potentiates it as an attractive therapeutic drug candidate that may complement existing treatment regimens to improve outcomes for PAH patients.

The complement system plays an essential role in defensive immune processes. However, dysregulated complement activation may turn this beneficial protective system into a destructive tissue-damaging villain, as shown in various inflammatory, autoimmune, and ischemic disorders [48]. Since as early as in the 1980s, Cooper JD et al. has established a complement-induced PH in sheep via infusion of plasma containing zymosan-activated complement [49–51], this indicates a direct role of complement in PAH. Moreover, it is becoming increasingly recognized that complement system activation and inflammation play important roles in the pathogenesis of PAH [52, 53], e.g., complement activation could promote endothelial dysfunction in PAH [54], and complement alternative pathway activation has been shown to be associated with PAH in lupus nephritis patients [55]. Furthermore, Bauer EM et al. have shown that significant C3 fragments accumulated in lung sections from idiopathic PAH patients as well as chronic hypoxia-induced PAH mice, and the indices of PAH got significantly ameliorated in C3 knockout mice [12]. Theoretically, C5 cleavage has been shown to take place in the absence of C3 by alternative mechanisms [56]. In the same study, they were unable to detect the C5 cleavage product C5a in plasma or lung tissue, which makes the C5 very unlikely a player in this experimental setting [12]. Recently, Frid et al. identified the activated alternative pathway as an essential regulator of hypoxia-induced proinflammatory and pro-proliferative changes in the lung [13]. Similar findings of alternative pathway activation were recently reported by high-throughput analysis of the plasma proteome in patients with PAH, in which increases in the activator of the alternative pathway, CFD, and decreases in the inhibitor CFH identified patients with PAH with high risk of mortality [57]. Fibroblast-released small extracellular vesicles served as critical mediators of complement-induced perivascular/microenvironmental inflammation in PH [15].

One study has shown that C3a increases ATP efflux from the monocyte cytosol and leads to subsequent increased activation of the ATP receptor P2X7, a potent Signal 2 for NLRP3 inflammasome activation [58]. In our current study, we applied the MCT-induced PAH rat model and supplemented the underlying mechanisms: C3a could promote subsequent inflammasome NLRP3 activation and release proinflammatory cytokines (IL-1β and IL-18), which resulted in vascular remodeling and finally pulmonary arteriole obstruction. One study has also shown that IL-1β promotes PASMC proliferation [34]. The exact origin of C3a is unknown in PAH, and we could not exclude the role of monocyte/macrophage-derived C3 in the lung tissues. A monocyte/macrophage-specific complement C3 knockout mouse/rat model is required in future studies.

The selective C3 inhibitor CP40 has been found therapeutically effective in various diseases [17, 20, 21, 23–27, 59], but has yet not been tested in the PAH model. In the current study, we applied the newly identified CP40 analog, CP40-KK, with two more lysine attached to the C terminus, in the MCT-induced PAH rat model. In comparison with the parental peptide CP40, the CP40-KK showed a drastic improvement in their solubility (> 200-fold) at physiological pH. Most importantly, a study in non-human primates indicated that CP40-KK was associated with a higher terminal half-life (∼fivefold), higher maximum concentration (∼twofold), and decreased apparent clearance (∼2.6-fold) when compared to CP40 [29]. Sahu et al. reported that compstatin can bind only primates but not rodent C3 in 2003[60], while our current study showed a potent effect of its analog CP40-KK in a rat PAH model. However, other groups have also shown that CP40 works in rodent models, e.g. CP40 can protect against choroidal neovascularization in a laser photocoagulation rat model [61], inhibit the activation of microglia in a bone cancer pain rat model [62], and ameliorate interstitial fibrosis in a unilateral ureteral obstruction mouse model [17]. Considering the evolutionary history of compstatin and our current knowledge, we speculate that the following two major aspects could result in the discrepancy: [1] the peptide structure, since the CP40-KK has been significantly modified in comparison with the compstatin peptide used in the assay 25 years ago. Generally speaking, modification of the amino acid could further change the 3-dimensional structure of the peptide and result in affinity variation in protein interaction; [2] Due to the different types of SPR instruments and complement C3 (full-length or cleaved fragments) differ across studies. Sometimes in vivo situation is not equal to the in vitro. Thus, an in vivo binding assay is required to further prove the hypothesis and intravital microscopy may be a good option.

## Conclusion

In the current study, we confirmed a direct role of the complement component C3 in the pathophysiological process of PAH and found that CP40-KK, a new analog of selective C3 inhibitor CP40, could protect against the established PAH in the MCT-induced rat model. These findings will shed new light on the PAH research and the treatment of this devastating disease.

### Supplementary Information

**Additional file 1: Table S1.** Reagents. **Figure S1.** Serum triglyceride and cholesterol levels in rat PAH models. **Figure S2.** The effect of CP40-KK on oxidative stress in the lungs of the PAH rats. **Figure S3.** The full blot image of cleaved caspase-1.

## Data Availability

The original contributions presented in the study are included in the article. Further inquiries can be directed to the corresponding author.

## Abbreviations

LV: Left ventricle
MCT: Monocrotaline
NLRP3: NOD-like receptor protein 3
PAH: Pulmonary arterial hypertension
PASMC: Pulmonary arterial smooth muscle cell
RVSP: Right ventricle systolic pressure
RV/(LV + S): Right ventricular/(Left ventricular + septum)
SPR: Surface plasmon resonance
