# Supplementary material for: A novel complement C3 inhibitor CP40-KK protects against experimental pulmonary arterial hypertension via an inflammasome NLRP3 associated pathway
Source: J Transl Med. 2024 Feb 16;22:164. doi: 10.1186/s12967-023-04741-z (PMC10870435; doi:10.1186/s12967-023-04741-z)
Supplement: Supplementary file 1 — Additional file 1: Table S1. Reagents. Figure S1. Serum triglyceride and cholesterol levels in rat PAH models. Figure S2. The effect of CP40-KK on oxidative stress in the lungs of the PAH rats. Figure S3. The full blot image of cleaved caspase-1. [file 12967_2023_4741_MOESM1_ESM.docx]

Additional file Materials for

**A novel complement C3 inhibitor CP40-KK protects against experimental pulmonary arterial hypertension *via* an inflammasome NLRP3 associated pathway**

**Lei Dai et al.**

**Corresponding authors: Hesong Zeng and Xiaodan Zhong**

**zenghs@tjh.tjmu.edu.cn and zhongxiaodan@hust.edu.cn**

The PDF file includes:

Reagents

Methods

Figs. S1 to S2

References

### Additional file Table 1：Reagents

| REAGENT or RESOURCE | SOURCE | IDENTIFIER |
| --- | --- | --- |
| Antibodies |  |  |
| Rabbit anti-α-Smooth Muscle Actin | Abclonal | Cat# A17910; |
| Rabbit anti-PCNA | Abclonal | Cat# A0264; |
| Rabbit anti-Caspase-1 | Abclonal | Cat# A0964; |
| Rabbit anti-IL1β | Abclonal | Cat# A16288; |
| Rabbit anti-IL18 | Abclonal | Cat# A16737; |
| Rabbit anti-Transferrin | Abclonal | Cat# A1448; |
| HRP Goat Anti-Mouse IgG (H+L) | Abclonal | Cat# AS003; |
| HRP Goat Anti-Rabbit IgG (H+L) | Abclonal | Cat# AS014; |
| Rabbit anti-NOX1 | Abclonal | Cat# A8527; |
| Rabbit anti-NF-kB p65 | Affinity Biosciences | Cat# AF5006; |
| Rabbit anti-phospho-NF-kB p65 (Ser536) | Affinity Biosciences | Cat# AF2006; |
| Rabbit anti-C3a | Biomatic | Cat# CAU28002; |
| Rabbit anti-SOD2 | Boster | Cat# BA4566 |
| Rabbit anti-NLRP3 (D4D8T) | Cell Signaling | Cat# 15101; |
| Rabbit anti-Cyclin D1 | Cell Signaling | Cat# 2922; |
| Mouse anti-β-Actin | Proteintech | Cat# 66009-1-Ig; |
| Rabbit anti-NOX4 | Proteintech | Cat# 14347-1-AP; |
| Mouse anti-gp91phox | Santa Cruz | Cat# sc-130543 |
| Mouse anti-Galectin 3 | Servicebio | Cat# GB12940 |
| CY3 Conjugated Goat anti-Rabbit IgG(H+L) | Servicebio | Cat# GB21303; |
| FITC Conjugated Goat anti-mouse IgG(H+L) | Servicebio | Cat# GB22301; |
| Chemicals, Peptides, and Recombinant Protein |  |  |
| Monocrotaline | Sigma-Aldrich | C2401 |
| CP40KK | GL Biochem Shanghai | dTyr-Ile-[Cys-Val-Trp(Me)-Gln-Asp-Trp-Sar-His-Arg-Cys]-mIle-Lys-Lys-NH2 |
| Complement C3/C3a Protein, Human | MedChemExpress | HY-P7862 |
| SB290157 trifluoroacetate | MedChemExpress | HY-101502A |
| IL-1RA | MedChemExpress | HY-P72566 |
|  |  |  |

### 2. Additional file methods

### 2.1 EdU Assay

Cells seeded in the 24-well plate were labeled with 10μmol/L of EdU (Meilun, Dalian, China) for 2 h at 37℃. Subsequently, cells were fixed in 4% of paraformaldehyde for 20 min and incubated in phosphate-buffered saline (PBS) containing 0.5% of Triton- X100 for 15 min. After being washed three times with PBS, 150μL of dying solution was applied per well for 30 min and nuclei staining with Hoechst 33342 for 10 min away from light. The proliferating cells were labeled with green fluorescence, with a maximum excitation wavelength of 491nm and a maximum emission wavelength of 518nm for 488-Azide. The ratio of EdU-positive cells was finally calculated. The proliferative rate = EdU-positive cells / Hoechst-positive cells.

### 2.2 Immunohistochemistry and immunofluorescence

After hemodynamic measurements, the lungs were perfused with cold phosphate-buffered saline (PBS) and fixed in 4% formaldehyde for 24 hours. The whole lungs were embedded in paraffin, and cross-sections (5μm) were prepared. Paraffin sections were used for immunostaining. For immunohistochemistry, lung sections were initially de-paraffinized, rehydrated, and boiled in a pressure cooker containing a citric acid buffer (pH 6.0) for 20 mins to retrieve antigens. Then the slides were blocked with 5% BSA for 1 h and incubated with primary antibodies overnight at 4°C and with corresponding secondary antibodies for 30 mins at room temperature. The primary antibody to αSMA (1:100, Abclonal) and Galectin 3 (1:100, Servicebio) was used. Immunolabels were detected with 3, 3-Diaminobenzidine (DAB), after which the nuclei were counterstained with hematoxylin. Slides were inspected under an optical microscope (Olympus BX61, Tokyo, Japan) at 200× and 400× magnification. For quantification analysis, the mean integrated optical density (IOD) for immunohistochemistry was determined using Image J software (National Institutes of Health, Bethesda, MD)^1^.

For immunofluorescence, the lung slides were prepared as described in histological analysis till the incubation of secondary antibodies. Corresponding fluorescently labeled secondary antibodies (anti-mouse IgG FITC: 1:200, anti-rabbit IgG-CY3 red: 1:200) were added for 60 mins and sections were rinsed twice in PBS. Slides were covered with vecta shield mounting medium containing nuclear stain DAPI (Vector Laboratories, CA, USA). Specimens were analyzed on a Leica SP5 confocal microscope^2^.

### 2.3 Western Blotting

Proteins were collected from the fresh frozen-rat lung tissues using RIPA lysis buffer. Protein concentration was quantified using a BCA reagent. Equal amounts of protein were electrophoretically separated on 10% SDS polyacrylamide gel, transferred to PVDF membranes, and probed with desired primary antibodies overnight at 4°C. Membranes were then washed with TBST and incubated with anti-mouse IgG (1:5, 000) or anti-rabbit IgG (1:5, 000) horseradish peroxidase conjugated antibodies as indicated. Blots were developed with the enhanced chemiluminescence system. To compare and quantify levels of proteins, the density of each band was measured using Image J software. Equal loading for total cell or tissue lysates was determined by β-Actin western blot^3,4^.

### 2.4 ELISA

CP40-KK and its control peptide were dissolved in coating buffer at different concentrations and then added to wells of ELISA plate as designed. The coated plate was incubated at 4°C overnight. After removing the coating buffer from the wells, the plate was washed using wash buffer 3 times. Then the plate was incubated with plasma with the indicated dilution at 37°C for 2h. Next, the plate was washed for 5 times and incubated with the C3 antibody (1:2000, MP biomedicals, Irvine, CA, USA) at 37°C for 2h. Then the plate was washed for 3 times and incubated with corresponding HRP conjugated secondary antibodies (1:2000, Abcam, Cambridge, UK) at 37° for 1h. Finally, TMB substrate was added to each well for the indicated time at 37°C and then the stop solution was added to stop the TMB reaction. OD values were measured at 450nm using a microplate reader (Bio-tek, Vermont, USA). The coating buffer, wash buffer, TMB substrate, and stop solution were all bought from Boster Biological Technology (Wuhan, China).

### 2.5 CH50 assay

Plasma samples for biological assays from rats were centrifuged and stored at -80°C until used, and the total functional complement was determined with a CH50 assay based on the modified method of Mayer. This assay involves determining the quantity of plasma needed to induce 50% lysis of sheep erythrocytes sensitized with antibody anti-sheep erythrocytes. The 50% hemolytic standard tube was made by adding 0.3ml 2% sheep erythrocytes (Chundubio, Wuhan, China) to 1.2ml distilled water and then diluting with equivoluminal veronal buffer saline (Leagene, Beijing, China). The sheep erythrocytes were sensitized by adding a designated volume of a predetermined concentration of rabbit hemolytic serum (Solarbio, Beijing, China) in veronal buffer saline. The plasma of rats was first prediluted in veronal buffer saline at 1/5, 1/10, and 1/20. At every dilution, ten different volumes (20, 30, 40, 50, 60, 70,80, 90, 100, and 0ul) of prediluted plasma of rats were firstly mixed with corresponding volumes (280, 270, 260, 250, 240, 230, 220, 210, 200 and 300ul) of veronal buffer saline in ten tubes and then all ten tubes were added 200ul sensitized sheep erythrocytes respectively. All tubes were incubated in a 37°C water bath for 30min and then centrifuged at 2000r/min for 10min. The OD values of supernatant liquid in the ten tubes were measured at 541 nm and compared with that of the 50% hemolytic standard tube to get the most similar value. The total hemolytic complement (CH50) = dilution ratio / the quantity of plasma needed to induce 50% lysis of sheep erythrocytes^5-7^.

### 2.6 Surface plasmon resonance (SPR)

1. **Materials and Buffer Preparation:** Sensor Chip COOH should be prepared before the experiment. Prepare all buffer containing Running Buffer HEPES (10mM HEPES,150mM NaCl,3mM EDTA, with 0.005%Tween-20, pH7.4), Immobilization Buffer (10mM Sodium Acetate, pH5.0), and Regeneration Buffer (10mM Glycine-HCl). 200μL EDC /NHS solution.
2. **Chip Preparation and Ligand Capturing**: Dilute the human complement C3(rat complement C3、mouse complement C3) to 62.5μg/mL in an immobilization buffer. The activator is prepared by mixing 400mM EDC and 100mM NHS immediately prior to injection. Then inject to sample human complement C3 (rat complement C3、mouse complement C3) at a flow rate of 20μL/min to reach a capture level of about 4800 RU. The chip is deactivated by 1M Ethanolamine hydrochloride at a flow rate of 20 μL/min for 240s.
3. **Running Analyte by multi-cycle method**: Dilute compstatin (CP40-KK) with the same running buffer to 5 concentrations. Compstatin (CP40-KK) is injected into the flow cell of the channel at a flow rate of 20μL/min for an association of 240s, followed by 480s dissociation. The association and dissociation processes are all handled in the running buffer. Repeat 5 cycles of analyte according to analyte concentrations in ascending order. After each cycle of interaction analysis, the sensor chip surface should be regenerated completely with 10mM Glycine-HCl as injection buffer at a flow rate of 100μL/min for 30s to remove the ligand and any bound analyte.
4. **Analysis:** The analysis software used in this experiment is: TraceDrawer (Ridgeview Instruments ab, Sweden), and was analyzed by the One To One analysis model.

### 2.7 Lipid Measurements

Blood samples were obtained from rats, and plasma lipid levels were measured with a total cholesterol assay kit and triglyceride assay kit (Nanjing Jiancheng Bioengineering Institute, Nanjing City, China). 2.5μL plasma or 2.5μL calibrator was added into 250μL working solution, and then incubated at 37 ℃ for 10 minutes. OD values were measured at 500nm using a microplate reader (Bio-tek, Vermont, USA).

### 3. Additional file Figure

**Additional file Figure S1**


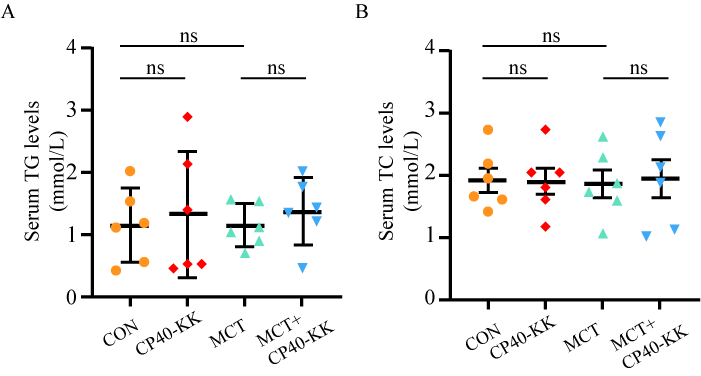


**Figure S1. Serum triglyceride and cholesterol levels in rat PAH models.**  Triglyceride(A) and cholesterol (B) levels were tested in rat plasma; mean ± SEM; Multiple comparisons made by one-way ANOVA.

**Additional file Figure S2**


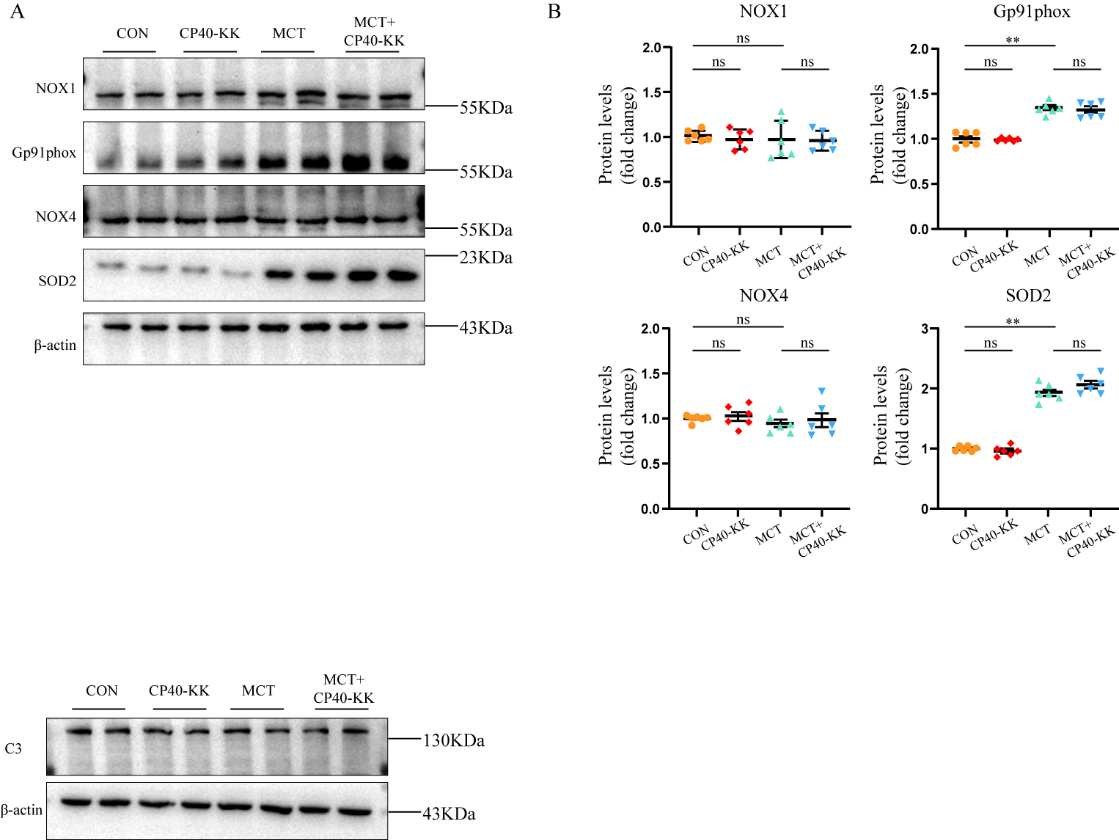


**Figure S2. The effect of CP40-KK on oxidative stress in the lungs of the PAH rats.** Representative Western blots (A) and quantification (B) of the NOX1, Gp91phox, NOX4 and SOD2 in lung homogenates of rat (B; n=6 per group).

**Additional file Figure S3**


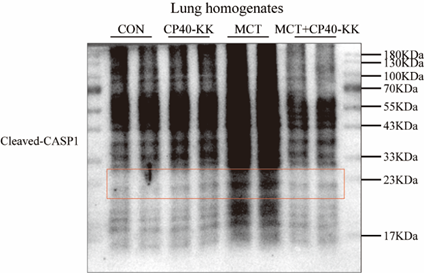


**Figure S3. The full blot image of Cleaved-CASP1**

**Reference**

1 Wang, T. *et al.* Weighted Gene Co-expression Network Analysis Identifies FKBP11 as a Key Regulator in Acute Aortic Dissection through a NF-kB Dependent Pathway. *Front Physiol* **8**, 1010, doi:10.3389/fphys.2017.01010 (2017).

2 Madhusudhan, T. *et al.* Defective podocyte insulin signalling through p85-XBP1 promotes ATF6-dependent maladaptive ER-stress response in diabetic nephropathy. *Nat Commun* **6**, 6496, doi:10.1038/ncomms7496 (2015).

3 Dong, W. *et al.* Activated Protein C Ameliorates Renal Ischemia-Reperfusion Injury by Restricting Y-Box Binding Protein-1 Ubiquitination. *J Am Soc Nephrol* **26**, 2789-2799, doi:10.1681/ASN.2014080846 (2015).

4 Wang, T. *et al.* Integrated bioinformatic analysis reveals YWHAB as a novel diagnostic biomarker for idiopathic pulmonary arterial hypertension. *Journal of cellular physiology* **234**, 6449-6462, doi:10.1002/jcp.27381 (2019).

5 Peffault de Latour, R. *et al.* Assessing complement blockade in patients with paroxysmal nocturnal hemoglobinuria receiving eculizumab. *Blood* **125**, 775-783, doi:10.1182/blood-2014-03-560540 (2015).

6 Ishizaki, J. *et al.* Low complements and high titre of anti-Sm antibody as predictors of histopathologically proven silent lupus nephritis without abnormal urinalysis in patients with systemic lupus erythematosus. *Rheumatology (Oxford)* **54**, 405-412, doi:10.1093/rheumatology/keu343 (2015).

7 Strong, W. M., Lea, D. J. & Ward, D. J. Measurement of total haemolytic complement activity in body fluids. *J. Clin. Pathol.* **31**, 527-530, doi:10.1136/jcp.31.6.527 (1978).
